# Machine learning identifies key individual and nation-level factors predicting climate-relevant beliefs and behaviors

**DOI:** 10.1038/s44168-025-00251-4

**Published:** 2025-05-08

**Authors:** Boryana Todorova, David Steyrl, Matthew J. Hornsey, Samuel Pearson, Cameron Brick, Florian Lange, Jay J. Van Bavel, Madalina Vlasceanu, Claus Lamm, Kimberly C. Doell

**Affiliations:** 1 Department of Cognition, Emotion, and Methods in Psychology, Faculty of Psychology, University of Vienna, Vienna, Austria; 2 Business School, University of Queensland, Brisbane, Australia; 3 Department of Psychology, University of Amsterdam, Amsterdam, Netherlands; 4 Behavioral Economics and Engineering Group, KU Leuven, Leuven, Belgium; 5 Department of Psychology, New York University; New York University, New York, USA; 6 Center for Neural Science, New York University; New York University, New York, USA; 7 Norwegian School of Economics, Bergen, Norway; 8 Department of Environmental Social Sciences, Stanford Doerr School of Sustainability, Stanford, CA 94305 USA; 9 Centre for the Advanced Study of Collective Behavior, University of Konstanz, Universitätsstraße 10, 78464 Konstanz, Germany

**Keywords:** Environmental social sciences, Climate-change mitigation, Climate-change policy, Psychology and behaviour, Psychology

## Abstract

While numerous studies have examined factors associated with climate-friendly beliefs and behaviors, a systematic, cross-national ranking of their key correlates is lacking. We use interpretable machine learning to quantify the extent to which different climate-relevant outcomes (climate change belief, policy support, willingness to share information on social media, and a pro-environmental behavioral task) are predictable and to rank 19 individual- and nation-level predictors in terms of their importance across 55 countries (*N* = 4635). We find notable differences in explained variance for the outcomes (e.g., 57% for climate change belief vs. 10% for pro-environmental behavior). Four predictors had consistent effects across all outcomes: environmentalist identity, trust in climate science, internal environmental motivation, and the Human Development Index. However, most of the predictors show divergent patterns, predicting some but not all outcomes or even having opposite effects. To better capture this complexity, future models should include multi-level factors and consider the different contexts (e.g., public vs private) in which climate-related cognition and action emerge.

## Introduction

Considering the detrimental impacts humans have on the environment and climate^1^, enormous effort has been dedicated to studying the predictors of climate-friendly beliefs and behaviors^2–5^. However, achieving a holistic portrait of which predictors are most important across various climate outcomes remains elusive. Much of the existing research has focused on individual-level factors, such as psychological traits or demographics^2,3^, and has been conducted in single, mostly Global North nations^6^. This is problematic as it limits the generalizability of the results, especially on an international scale, and it limits the ability to identify influences that operate at the national level.

Research has revealed that an overreliance on populations from the Global North has constrained our understanding of environmental attitudes and behaviors. Studies indicate that findings derived from these populations often do not generalize to the diverse cultural, socioeconomic, and political contexts present globally^6,7^. This narrow focus risks perpetuating biased theoretical models and intervention strategies that overlook the unique environmental challenges and perspectives in non-Western settings. Expanding research beyond Global North samples is therefore essential to ensure that environmental psychology reflects the full complexity of the different factors driving people’s climate change beliefs and behaviors.

While individual factors undoubtedly contribute to shaping climate-related attitudes and behaviors, individuals do not act in a vacuum. Rather, they exist within broader social, political, and economic structures that shape both perceptions of climate change and their ability to engage in pro-environmental behavior. Relatedly, cross-national differences in climate change beliefs and behaviors are well-documented^8–10^. For example, the percentage of people who consider climate change a “very serious threat” varies substantially across countries, from 69% in Italy to 13% in Egypt^11^. Such differences suggest that national-level factors—such as a country’s level of development, inequality, or fossil fuel reliance—may play a critical role in shaping climate-related outcomes. However, less is known about which nation-level factors are most relevant, although a growing body of research has explored some factors such as nation-level GDP, education, and per capita carbon emissions^4,12–18^ – which may help clarify how broader socioeconomic contexts shape responses to climate change. Thus, including both individual and nation-level factors offers a more comprehensive framework to understand and address the complex drivers of climate change beliefs and behaviors.

Moreover, when it comes to studying individual pro-environmental behavior, few studies have focused on actual, consequential behavior^19–21^ as opposed to attitudes, intentions, and responses to hypothetical scenarios. Given a convergence of evidence that people’s climate actions are often discrepant from what they self-report^19,20,22^ – the disproportionate focus on non-behavioral measures risks skewing our perception of the most relevant predictors and thus potentially identifying the wrong targets for climate action.

Furthermore, investigating multiple climate-change–relevant outcomes is crucial because each outcome may be influenced by distinct sets of factors, and focusing solely on one may obscure the importance of others. For example, the factors that predict people’s belief in climate change might differ from those that predict their willingness to engage in effortful climate actions. By examining and comparing several outcomes, we can identify whether certain factors consistently matter across different climate-relevant outcomes or if some factors uniquely predict certain attitudes and behaviors.

To overcome these shortcomings and to obtain a bird’s-eye view of the most important predictors of several aspects of climate action – one that is both multi-national (in terms of sampling) and multi-dimensional (in terms of the range of attitudes and behaviors canvassed), we argue for a need to compare multi-level predictors of different climate-relevant outcomes within the same international dataset. The goal of this work was to 1) quantify how predictable different climate-relevant outcomes are and 2) obtain an omnibus test of the relative importance of various predictors. Using an advanced machine-learning approach that is relatively novel for environmental social science, we rank-ordered 19 individual and nation-level predictors across four outcomes to create a comprehensive portrait of their relationships. Ranking the predictors is essential because it helps identify the most influential factors to the outcomes of interest. In the context of machine learning models, ranking predictors allows us to understand which variables have the greatest impact on the model’s predictions. This information is valuable for understanding the relative influence of different factors and can help guide decisions on where to focus future research and inform the design of interventions for the respective outcome of interest. For example, knowing which psychological or demographic factors are most strongly associated with climate-related beliefs and behaviors can be a first step in helping to tailor communication strategies.

**Fig. 1:**
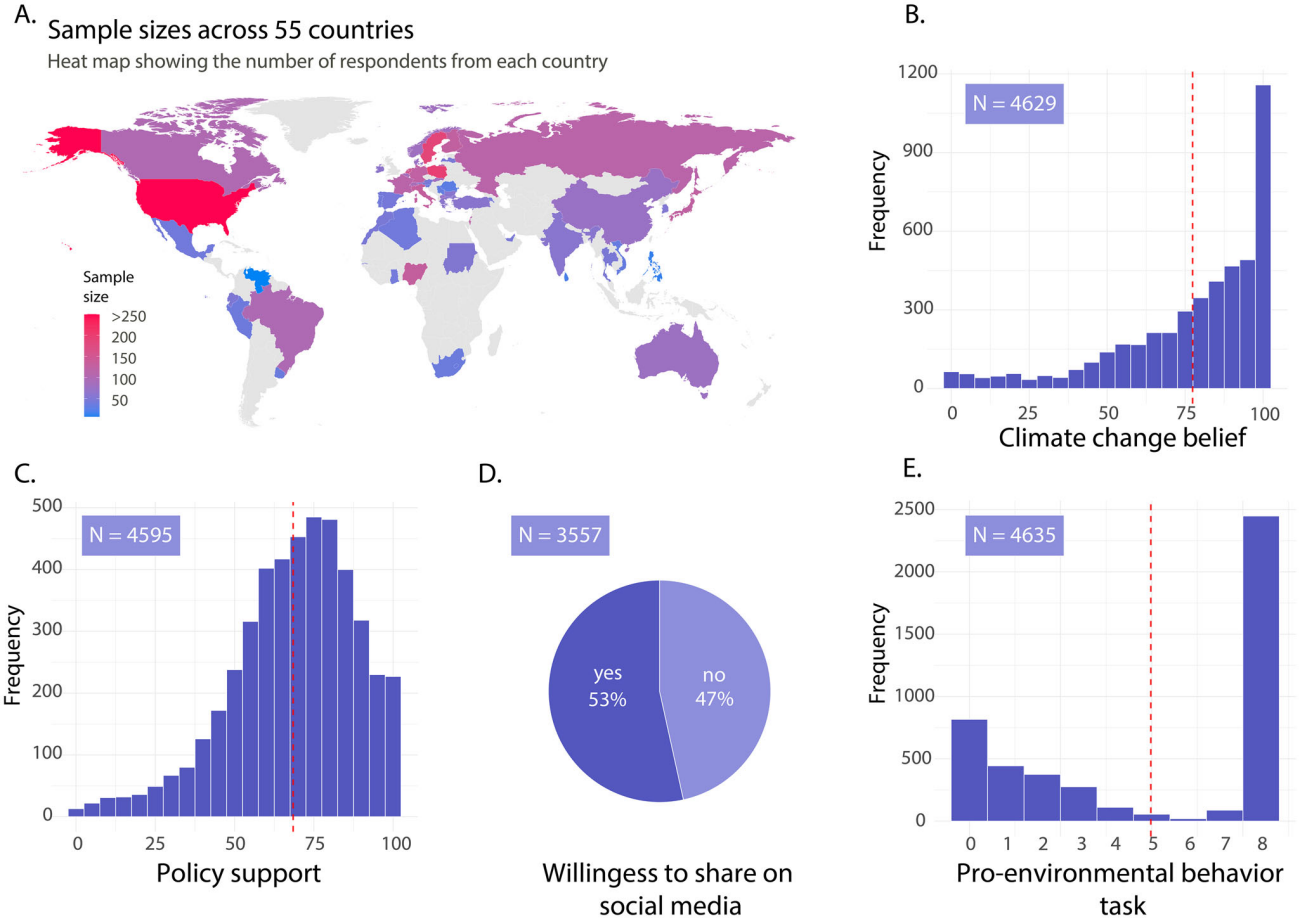
Overview of the countries, sample sizes, and distributions of outcome measures. **A** Heatmap showing the sample size in each country. Sample size and distribution of **B** climate change belief (*M* = 77.36, *SD* = 24.74), **C** policy support (*M* = 68.46, *SD* = 19.79), **D** willingness to share on social media, **E** number of completed pages of the pro-environmental behavior task (*M* = 4.98, *SD* = 3.43). The red dotted vertical line on plots (**B**, **C**, & **E**) indicates the mean value.

To achieve these goals, we analyzed data collected as part of the International Climate Psychology Collaboration^23^ (ICPC; *N* = 4635; see Fig. 1A and Supplement Table S1). The outcome measures included climate change belief, climate policy support, willingness to share climate-relevant information on social media, and a pro-environmental behavior task (adapted version of the Work for Environmental Protection Task^24^; Fig. 1B-1E shows the outcome distributions). This task was an online behavioral measure that mimics the trade-off between time-consuming effort and positive environmental impact typical for many sustainability-related decisions in daily life. Participants could voluntarily complete pages of a tedious, cognitively demanding task in exchange for one tree planted per page. For the predictors, we included individual-level demographic variables and psychological antecedents from the ICPC dataset, and we supplemented data on nation-level factors from additional sources. The full list of predictors and reasoning for inclusion are shown in Table 1, and their distributions can be found in the supplementary information (Fig. S1).

**Table 1:** Description of the predictors included in each of the models

	Predictors	Reasoning for inclusion
**Demographics**	Age	Demographics have been identified as relevant factors for some climate-relevant outcomes in the literature, albeit with generally small effect sizes^2,3^. These findings can be useful for selecting groups that should be particularly targeted with communication and intervention strategies.
	Gender^a^	
	Education	
	Political orientation	
	Income	
	MacArthur's subjective social status	
**Psychological antecedents**	Trust in climate science/ scientists	As climate risks are not always directly experienced, trusting experts is important to evaluate these risks and act accordingly. Research has shown that trust in climate scientists relates to different types of pro-environmental behaviors^64,65^.
	Trust in government	Governments have a crucial role in addressing climate change by managing risks, implementing policies, and fairly managing revenues from climate policies, making trust in governments a potentially relevant factor. It has been shown to correlate more strongly with public climate-relevant behaviors than private^65^.
	Environmentalist identity	Social identity theory suggests people’s behavior is shaped by the social groups with which they identify (i.e., environmentalists, humanitarians, or global citizens), and the degree of this identification should relate to both public and private environmental action^37–39,51^.
	Humanitarian identity	
	Global citizen identity	
	Environmental motivation: internal	According to self-determination theory, there are two main types of motivation driving our behavior - intrinsic and extrinsic^52^ - potentially having differential impacts on people’s behavior.
	Environmental motivation: external	
	Perceived scientific consensus on climate change	The role of perceived scientific consensus has been identified as crucial, and misperceptions of scientific consensus have been associated with reduced levels of support for climate policies^66^^,67^.
	Perceived climate change belief in own country	The perceived belief in climate change of others is a measure of descriptive social norms, identified as crucial for behavior by multiple theories in social psychology (e.g., social identity theory^68^, value-belief-norm theory^69^). People underestimate the prevalence of pro-climate views of others^70,71^, which can be a barrier to climate change mitigation^72^.
**Nation level**	GINI coefficient	The GINI coefficient is a nation-level measure of income inequality. Inequality has been shown to fuel emission-intensive consumption and production and enable wealthy elites to obstruct climate policies^73^.
	Human Development Index (HDI)	HDI is a metric of development combining GDP per capita, education levels, and life expectancy. The more economically developed a nation, the less likely they are to show climate change concern^50^ and to invest part of their income to mitigate climate change^4^.
	Climate risk index	There is mixed evidence that personal experience with extreme weather can engage people with climate. Some papers suggest extreme weather events such as floods can increase belief in climate change^74^ while others say there is no relationship^75^.
	Carbon emissions per capita	People from countries relying heavily on fossil fuels are less willing to acknowledge the risk of climate change^12^, and research has shown an association between climate skepticism and carbon emissions.

^a^Due to restrictions by the ethics boards in some countries, the item was modified to include only two options (male and female).

We employed an interpretable machine learning approach based on gradient-boosted decision trees (GBDT) to assess the relative importance of predictor variables in our model. GBDT constructs an ensemble of decision trees sequentially, where each new tree corrects errors from the previous ones, optimizing predictive accuracy while maintaining robustness to overfitting. By running a “competition” amongst variables, we systematically evaluated their influence, allowing us to identify the most important predictors in a transparent and data-driven manner. Throughout the manuscript, we refer to “predictor importance” as defined within the SHAP framework^25^. Here, importance relates to how much the prediction of a model changes (compared to the average model output) due to a specific predictor.

The selected analysis approach balances predictive performance with interpretability, enabling us to extract meaningful insights from complex relationships within the data and was chosen due to the numerous advantages it has over more traditional regression-based models^26–29^. First, by modeling non-linear relationships, it provides more comprehensive and realistic information about the associations between predictors and outcomes^26^. Here, regression-based models would likely miss predictors with non-linear effects (see SI Fig. S2 for a correlation matrix). Second, it is not dependent on restrictive assumptions that often bias traditional statistical methods and thus allows us to examine many variables, even correlated ones, simultaneously^27–30^. Third, this approach focuses on predictive accuracy, achieved by leveraging nested cross-validation, allowing us to assess how the predictions generalize to independent data^31^. Last, for each of the four outcome measures, this approach allowed us to rank-order the predictors in terms of their ability to explain change in each outcome variable. We preregistered our predictors and analyses using the “secondary data template” (https://osf.io/6jc85).

## Results

### Model fit of the different climate-relevant outcomes

First, we investigated how much of the variance in the different outcomes can be explained by the predictors by looking at each model’s fit (using R^2^ for the quasi/continuous variables and classification accuracy for the binary willingness to share variable). The model explained different amounts of variance for each of the outcomes: climate change beliefs (57%), climate policy support (46%), willingness to share (74% classification accuracy), and pro-environmental behavior (10%).

### Interpreting the relative predictive value of each variable

**Fig. 2:**
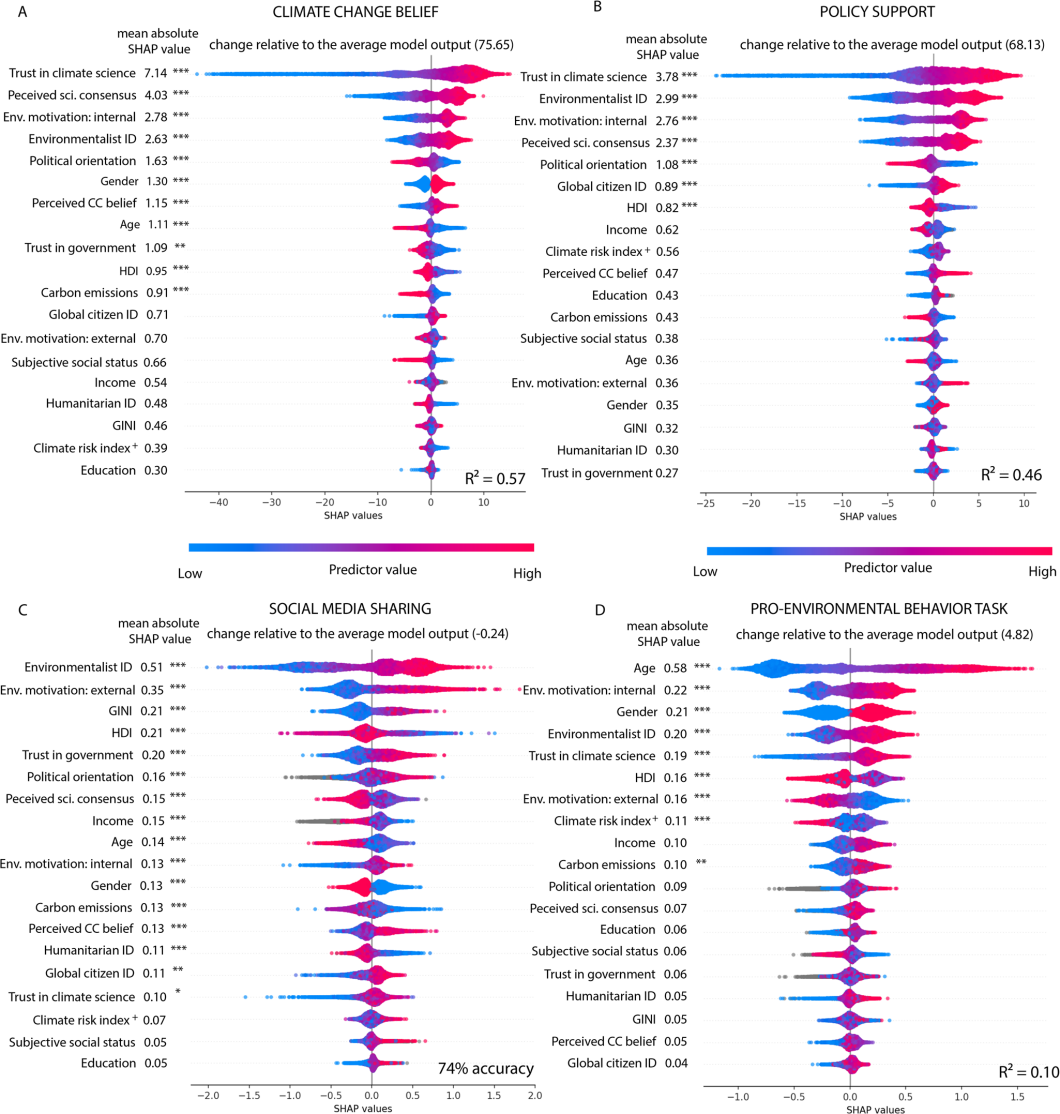
Summary plots for the effects of the predictors on the four different outcomes. **A** climate change belief, **B** policy support, **C** willingness to share on social media, and **D** pro-environmental behavior task. Each subplot depicts the predictors, ranked from highest to lowest importance for the model output. Each dot corresponds to the SHAP value of an individual prediction example. Pink color indicates higher predictor values, blue lower ones, and gray corresponds to missing values. SHAP values indicate how much the current output of the model deviates from the average model output due to a specific predictor value. A negative SHAP value (extending to the left of the vertical line) indicates a decrease in the outcome measure (or its log odds), while a positive one (extending to the right) indicates an increase in the outcome measure (or its log odds). The absolute mean SHAP value is indicated next to the predictor names on the left of each plot. Significance marked as follows: *** *p* < 0.001, ** *p* < 0.01, * *p* < 0.05. Significance is compared to null hypothesis data obtained from models trained and tested on shuffled data. No multiple comparison corrections were applied. Note: Gender was coded such that male = 0, female = 1; political orientation high scores = more conservative, low scores = more liberal; + climate risk index high scores indicate lower risk, and low scores higher risk. CC = climate change, ID = identity, HDI = human development index, GINI = income inequality coefficient.

To analyze the importance of each predictor, we used SHaply Additive ExPlanation (SHAP) values^25^, which assesses the importance of each predictor in driving the change in each outcome variable, considering interaction effects. It describes how much the prediction of a model changes (compared to the average model output) due to a specific predictor value. A SHAP value of 0 suggests that the predictor value has no effect on the outcome measure. A positive or negative value indicates an increase or decrease respectively in the outcome measure. For example, as shown in Fig. 2A, the top predictor for climate change belief was trust in climate science. Each colored dot corresponds to one individual, with pink dots indicating high trust and blue dots indicating low. Values on the left side of the vertical line (i.e., negative SHAP values) predict the outcome negatively, while values on the right (i.e., positive SHAP values) predict the outcome positively. Therefore, low trust (indicated by the blue dots) was a strong predictor of low climate change belief (indicated by large negative SHAP values), and high trust (indicated by the pink dots) was a positive predictor of high climate change belief.

To rank-order the predictors’ relative importance, we calculated each predictor’s absolute mean SHAP value (i.e., we aggregated these individual local changes to obtain a global measure of the predictor’s importance). Higher mean absolute SHAP values imply larger contributions to each outcome measure^25^ and correspond to the mean change in the outcome measure after including the predictor in the model. For example, the mean absolute SHAP value for trust in science in the belief model is 7.14. As the outcome measure (i.e., climate change belief) is assessed on a continuous 0-100 scale, this value indicates that, on average, trust in climate science contributes to a change of 7.14 points in climate change belief (relative to the average model output of 75.65). Policy support was measured on the same 0-100 scale, and therefore, trust in climate science with a mean absolute SHAP value of 3.78 contributes to an average change of 3.78 points (relative to the average model output, 68.13; Fig. 2B).

For the willingness to share information on social media, measured via a single choice item, we included only the participants who said yes or no (excluding the ones who report not using social media; Fig. 2C). For this model, mean absolute SHAP values indicate the average absolute change in the log odds. The top-ranking predictor, environmental identity, had a mean absolute SHAP of 0.51, contributing to a 0.51 change in the log odds for the outcome (relative to the average model output of -0.24 log odds). For the pro-environmental behavior, in which the outcome was the number of completed pages (0 to 8), the mean absolute SHAP values indicate the mean change in the number of completed pages. The top-ranking predictor for the task was age, with a mean absolute SHAP value of 0.58, indicating that age contributed to an average change of 0.58 completed pages (relative to the average model output of 4.82 pages). As can be inferred from the color coding in Fig. 2D, younger people completed fewer pages, and older people completed more (largely linear effect).

### Predictors with consistent effects across all outcomes

**Fig. 3:**
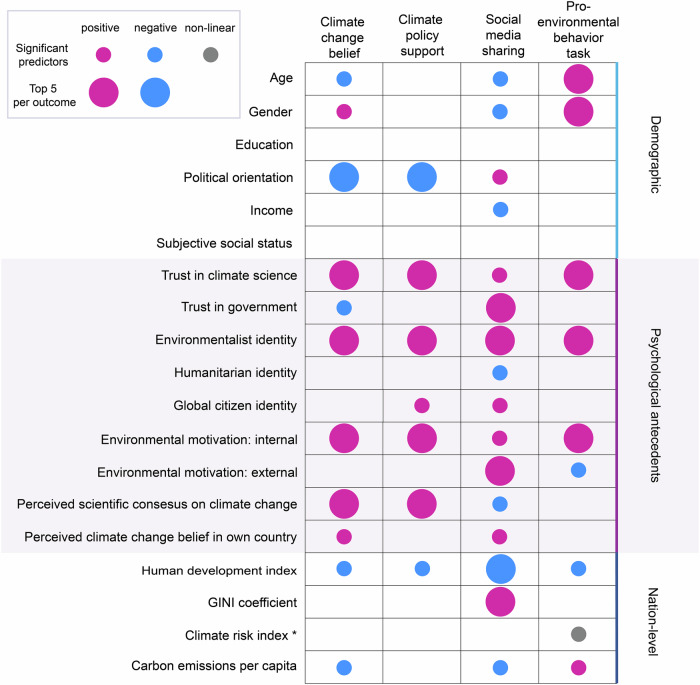
Schematic representation of the overall direction of the significant effects of all the predictors on the different outcomes. Small circles represent predictors that have a significant effect on the outcome, while big circles represent significant predictors that are the top 5 most important predictors for each outcome. The color of the circle indicates the direction of the relationship between the predictor and the outcome. If a predictor had a positive effect (i.e., a higher predictor value is associated with a higher outcome value), it is marked in pink, and if it had a negative, it is in blue. Effects that did not show an overall positive or negative direction were labeled as non-linear and marked in grey. Note: * climate risk index: high scores = low risk, low scores = high risk; gender: male 0, female 1; political orientation: high scores = conservative, low scores = liberal; GINI: income inequality coefficient.

Four of the 19 predictors exhibited consistent effects across all four climate-relevant outcomes (Fig. 3): environmentalist identity, trust in climate science, internal environmental motivation, and HDI. Among the psychological antecedents, environmentalist identity was one of the five strongest predictors and was positively related to all four outcomes (all *p*s < 0.001). Further predictors with consistent positive effects were higher trust in climate science (all *p*s < 0.001, except willingness to share *p* = 0.028) and higher internal environmental motivation (all *p*s < 0.001).

The only nation-level predictor showing consistent effects was HDI, whereby participants from countries with higher HDI had lower scores on all outcomes (all *p*s < 0.001). There were no consistent demographic predictors.

### Predictors with divergent effects across outcomes

Most predictors had divergent effects across the four outcome measures (see Fig. 3). For example, while left/liberal political orientation predicted higher climate change belief and higher climate policy support (both *p*s < 0.001), it negatively predicted willingness to share (*p* < 0.001) and was unrelated to pro-environmental behavior (*p* = 0.391). Perception of scientific consensus on climate change was one of the top five positive predictors for climate change beliefs (both *p*s < 0.001). However, it had a negative impact on willingness to share (*p* < 0.001) and no significant effect on the pro-environmental behavior (*p* = 0.370). External environmental motivation did not have a significant effect on climate change belief (*p* = 0.345) or policy support (*p* = 0.055), but it was among the top five positive predictors for willingness to share (*p* < 0.001), and it had a negative effect on the pro-environmental behavior (*p* < 0.001). Per capita carbon emissions had a negative effect on climate change belief and willingness to share (both *p*s < 0.001), a positive effect on the pro-environmental behavior (*p* = 0.006), but no significant effect on policy support (*p* = 0.406). Additional plots for the predictors and outcomes can be found on OSF (https://osf.io/m5uw7/files/osfstorage).

### Interactions among predictors

All first-order interactions (171 per model) were relatively unimportant based on SHAP values compared to the main effects and are therefore reported in the SI Figures S3-S6, and plots for each interaction are uploaded on the OSF (https://osf.io/m5uw7/files/osfstorage).

## Discussion

Climate change presents a global challenge that requires a comprehensive understanding of the factors that shape public beliefs and behaviors. In this study, we rank-ordered 19 individual- and nation-level predictors of climate-relevant outcomes across 55 countries using state-of-the-art interpretable machine learning modeling. Only four predictors showed consistent effects across all outcomes, presenting promising targets for refining future models of climate change perception and action. This includes three psychological antecedents – environmental identity, trust in climate science, and internal environmental motivation – and one nation-level predictor – HDI. The other 15 predictors showed divergent patterns, predicting some but not all outcomes and/or having opposite effects across outcomes.

The first goal of this analysis was to investigate the predictability of the different climate-relevant outcomes. We showed that there were substantial differences in the explained variance/classification accuracy across the four outcomes, with a notably low explained variance for effortful private pro-environmental behavior. While the other models had a relatively good fit (e.g., 57% of the variance was explained in the climate change belief model and 46% in the policy support), the same predictors explained only 10% of the variance associated with the pro-environmental behavior task. Relatedly, a recent study found that psychological and demographic factors explained 62% of the variance in self-reported sustainable clothing consumption but only 12% of the actual number of clothes purchased^32^. One explanation might be that people’s general pro-environmental tendencies do not always translate directly into behavior, especially when costs are high^33,34^ (but see also^35^). Another explanation is that the effortful behavior we observed may be influenced by unmeasured factors, such as current cognitive state or contextual elements like time availability, rather than the interindividual differences assessed. For example, participants knew in advance that they could complete a maximum of eight pages (in exchange for us planting one tree per completed page), and half of the sample completed all pages. We speculate that a kind of implementation intention/commitment setting^36^ effect might have overridden the measured individual- and nation-level predispositions. Multiple other studies have shown that objective behavior is hard to predict^20,32^, and situational factors like task boundaries or prior commitment may sometimes exert a stronger influence than underlying predispositions. Together, these findings highlight the need to investigate how contextual and cognitive factors shape behavior, particularly when specific goals or limits are communicated in advance.

Our second overarching aim was to identify the most important predictors for our measured outcomes. Environmentalist identity emerged as one of the top predictors across all outcomes. A large amount of research has focused on studying the relationship between different kinds of identities and climate action^37–39^. Our findings highlight that environmentalist identity was among the most consistent predictors across outcomes. In a next step, researchers could explore a potential causal route linking environmentalist identity to climate-relevant behaviors, particularly given its relative importance compared to other related identities, such as humanitarian and global citizenship. For example, studies might assess the effectiveness of educational programs that encourage participation in local conservation projects using randomized controlled designs to determine their impact on strengthening environmentalist identity and fostering climate-friendly beliefs and behaviors.

Another top predictor was trust in climate science and climate scientists. As climate change is not easily observable, people rely on institutions they trust for accurate information. However, this trust is fragile and can be undermined by well-funded counter-movements (e.g., fossil fuel lobbyists^40^). To prevent the systematic erosion of trust, researchers are exploring strategies like inoculation interventions to reduce people’s susceptibility to disinformation^41^, as disinformation campaigns frequently target scientists and scientific institutions to undermine their credibility. Future work could examine whether trust in climate science bolsters metacognitive confidence, i.e., confidence in the reliability of one’s own judgments about climate risks^42,43^. This enhanced metacognitive confidence may, in turn, support more effective questioning of misinformation and promote the search for accurate, trustworthy information, thereby providing a mechanistic link between trust in science and climate action.

Additionally, internal environmental motivation was positively related to all four outcomes and was a top predictor for all but the willingness to share on social media variable (a public but low-effort gesture). In contrast, high external motivation was a top positive predictor for willingness to share but it predicted the effortful, private behavior negatively. This pattern underscores a key aspect of self-determination theory, which distinguishes between external and internal motivation depending on the type of behavior. While external motivation may drive public actions, it can undermine intrinsic drive in private, effortful tasks. These results further support theoretical accounts categorizing private-sphere and public-sphere behaviors as distinct^44,45^. Public and private behaviors seem to be governed by distinct motivational and contextual factors, with public behaviors potentially benefiting from social influence strategies, while private behaviors might be more effectively influenced by fostering internal motivation. Together, these findings further highlight the utility of self-determination theory in understanding distinct types of climate-relevant behaviors.

Our results both replicate findings from previous research, which has been conducted in mostly Global North contexts and highlight important differences. For example, political orientation was one of the top 5 most important predictors for both climate change belief and policy support, such that being more liberal was related to higher climate change belief and support for policies. This is highly consistent with a large body of research in this domain^2,3,46^. However, political orientation had the opposite impact on willingness to share on social media (i.e., conservative participants were more willing to share) and had no significant impact on the pro-environmental behavior task. Thus, there seems to be consistent polarization of climate change at the conceptual/psychological level (i.e., beliefs and policy support) in line with previous research but not at the behavioral level (i.e., for public and private climate action); see also^47^ for in-depth investigation of the role of political orientation for these outcomes. These results highlight an important gap suggesting we need a better understanding of when and how political orientation predicts various forms of climate engagement and in cross-national, cross-cultural contexts. Given the focus on polarization in climate change, addressing this gap should be a top priority for researchers. A better understanding of these dynamics is important for developing targeted strategies to bridge divides, foster consensus, and facilitate more widespread climate action, particularly in regions where polarization is strongest^15,47,48^.

None of the demographic variables showed consistent effects across the outcomes, and their contributions to the model prediction were rarely large. Nevertheless, age emerged as the top predictor for the pro-environmental behavior task, with younger participants putting less effort in the task. Interestingly, a large body of literature^2,3^, and our present findings here for climate change belief and social media sharing point towards the opposite pattern for age (i.e., younger people being more environmentally conscious). We could speculate that older adults may demonstrate higher levels of conscientiousness and persistence in pro-environmental tasks requiring sustained effort – a pattern observed in research on prosocial behavior^49^. However, given that this model explained only 10% of the variance, this effect warrants cautious interpretation. Overall, our findings suggest that psychological predictors, such as environmentalist identity and trust in climate science, show more consistent relationships to climate-relevant outcomes than demographic factors. This may indicate that psychological variables could be more flexible targets for future interventions, though further examination is needed to explore their potential causal effects.

By leveraging a diverse, cross-national sample, we were able to not only assess individual-level predictors but also to examine nation-level influences. For instance, participants from countries with higher HDI showed lower scores in our climate-relevant outcomes. Similarly, studies have shown people from countries with higher per capita GDP (a component of HDI) were less likely to show climate change awareness and perceived risk^50^ and less willing to contribute part of their income to mitigate climate change^4^. One potential explanation is that countries with lower HDI/GDP do not have the same ability to buffer against the negative effects of climate change and are thus more sensitive to the need for action^13^. The effects of the other nation-level factors were divergent. For example, GINI (income inequality coefficient) was among the top five predictors for willingness to share on social media, while it had no significant association with any of the other outcomes. We speculate that inequality in a country could affect people’s willingness to be vocal about the need for a change, but not their private beliefs and behaviors. Together, this suggests that economic and social conditions at the national level can shape public engagement with climate issues in complex ways.

While some theories have recognized the role of some of our top predictors, like social identity theory highlighting the role of identity^51^ and self-determination theory incorporating different kinds of motivation^52^, the majority do not include these explicitly. It is even rarer for theories to incorporate nation-level influences next to individual ones despite increasing evidence for their importance^4,12,50^. Systematic differences in climate change attitudes and behaviors between countries have been observed for years^8,9^, but empirical and theoretical work on why that is the case has been scarce. We suggest that integrating psychological and sociological theory would be imperative for a better understanding of these phenomena, and including these key predictors will help build more comprehensive models that account for multi-level influences.

A number of considerations should be kept in mind when interpreting these results. First, the reported findings are associations, and we cannot assume a causal relationship between our variables. Thus, more dedicated research is needed to establish whether these factors can be causally linked to different aspects of climate change mitigation. Relatedly, we note that we defined the importance of predictors based on their effect sizes as determined by our machine learning framework. However, establishing real-world importance requires considering not only effect sizes but also the modifiability of predictors, meaning that some variables deemed less important by our framework might actually be more feasible targets for future investigations and interventions. Second, while the pro-environmental behavior task mimics the trade-offs that are naturally inherent in many pro-environmental behaviors^24^, it is still a very specific form of online behavior. This may also partially explain why the explained variance for this outcome was the lowest. Third, small sample sizes from certain countries and lack of representativeness should also be noted. However, despite these limitations, our sample is more heterogeneous than many other studies in this area, allowing for a more nuanced understanding of the interplay of these factors. Forth, while the interactions between the predictors were less important (based on their SHAP values) than the main effects and were only included in the supplement of this manuscript, we believe they provide a useful resource for potentially interested researchers who can use these to generate hypotheses that can be then tested independently. Lastly, the focus of the current work was to identify predictors that show consistent effects across a diverse sample, however, prior research has indicated that the effects of individual level predictors can have differential impacts in different regions^48^. Thus, a potential future direction could be to investigate how the effects of these predictors of climate-related beliefs and behaviors vary across different regions.

To conclude, our findings yield several key insights. First, while established predictors from environmental social science explained substantial variance in most climate-relevant outcomes, they accounted for considerably less variance in the effortful pro-environmental behavior. Second, more so than demographic factors, psychological factors such as trust in climate science and national factors like the Human Development Index were shown to be robust predictors of public responses to climate change. Lastly, the variability in the ranking and direction of the majority of the predictors underscores the importance of including multiple outcome variables in future research^10^ and considering the different contexts (e.g., public vs private) in which behaviors happen.

## Methods

### Sample

We used data from the International Climate Psychology Collaboration (ICPC)^23^. The full dataset from the ICPC includes 11 intervention groups and a no-intervention control group. For the current analysis, we focused on the control group, as it was the only group that included data on psychological antecedents. The final sample from the control group that we used consisted of *N* = 4635 participants from 55 countries (*M* age = 39.07 ± 15.69, 52*%* women). The included countries and the sample sizes can be seen in Fig. 1A and in the supplement in Table S1. Data from eight countries from the original dataset could not be included due to missing values (see below for more information). The sample size varies across each model (due to missing values in some of the outcome measures). For climate change belief, it was *N* = 4629; for policy support, *N* = 4595; for willingness to share on social media, *N* = 3557 (here we also excluded the people who said they do not use social media); and the pro-environmental behavior task *N* = 4635.

The full sample (including all the intervention groups and the control group) was predominantly collected using market research companies: 74% of the sample was matched for at least one demographic variable, and 65% was matched for both age and gender. As we are using a subsample of the full dataset (i.e., only the control group), we cannot claim our sample was nationally representative for demographic or socioeconomic indices. In the ICPC project, country selection was based on a global call for collaboration via social media, personal networks, and mailing lists, inviting research teams from around the world to contribute data. Rather than using a predetermined sampling method to represent every world region, the process was driven by the availability and willingness of potential collaborators. In addition, the organizational team sponsored additional data collection in underrepresented countries, including Morocco, Sudan, and Ecuador. This pragmatic strategy was designed to collect data from as many culturally and socioeconomically diverse settings as possible within the constraints of this type of collaborative effort.

#### Statistical power

There are still no established standards for conducting power analyses for complex, high-dimensional, multivariable models of the sort we used in the current analysis^53^. Because we are conducting a secondary analysis of an existing dataset, we followed three published conventions to determine whether our sample (*N* = 4635) was sufficiently powerful to test our questions. One simple suggestion is that 50 samples are required to start any meaningful machine learning based data analysis^54^. Another (more controversial) suggestion is that 10 to 20 participants per degree of freedom (predictor) is reasonable, which would lead to a total number of 190 to 380 participants required in the current case (with 19 predictors)^55,56^. Third, a conventional power analysis, employing a multiple regression with 19 predictors with an alpha of 0.05 and a power of 0.8, indicated that 787 samples would be necessary to detect small effects (Cohen’s d = 0.2). The current sample and design meets, and generally exceeds, all of these criteria.

### Procedure

The layout of the acquisition procedure for the ICPC dataset is described in detail in the ICPC data descriptor paper^23^. The procedure in the no-intervention control group we used for this analysis was as follows: Participants were presented with a definition of climate change (“Climate change is the phenomenon describing the fact that the world’s average temperature has been increasing over the past 150 years and will likely be increasing more in the future.”) and were subsequently exposed to the climate-relevant outcomes: climate change beliefs, climate policy support, and willingness to share a climate-relevant post on their social media (in a random order). Next, they were invited to engage in the pro-environmental behavior task, which was always the last outcome measure to be shown. Last, participants were asked to complete a series of psychological variables and demographic information. The data were collected between July 2022 and July 2023. Following the completion of data collection, we collected information on nation-level factors from additional sources for the countries included in the ICPC.

### Measures

The exact items used for the individual-level predictors and the outcome measures are fully described in an ICPC data descriptor paper^23^ and the full questionnaire items can also be found in our supplementary information (SI) in the supplemental methods section. Below we provide a brief summary.

#### Outcome measures

##### Climate change belief

Four items on a scale 0 = Not at all accurate to 100 = Extremely accurate (e.g., “Climate change poses a serious threat to humanity”; α = 0.93; see Fig. 1B for the distribution of responses and supplementary methods for the items).

##### Climate policy support

Nine items on a scale from 0 = Not at all to 100 = Very much so (e.g., “I support increasing taxes on airline companies to offset carbon emissions”, α = 0.88; see Fig. 1C for the distribution and supplementary methods for the items).

##### Willingness to share on social media

Participants first saw the text, “Did you know that removing meat and dairy for only two out of three meals per day could decrease food-related carbon emissions by 60%? It is an easy way to fight #ClimateChange #ManyLabsClimate, source: https://econ.st/3qjvOnn”. Subsequently they had to respond to a single choice item: “Are you willing to share this information on your social media?” with the following answer options: “Yes, I am willing to share this information”, “I am not willing to share this information”, and “I do not use social media” (see Fig. 1D for the distribution).

##### Pro-environmental behavior task

This task is an adapted version of the Work for Environmental Protection Task^24^ (WEPT) and is a multi-trial web procedure where participants are given the opportunity to engage in voluntary cognitive effort (screening numerical stimuli) in exchange for making donations to an environmental organization that plants trees. Participants were instructed to identify specific target numbers for which the first digit is even and the second digit is odd. They were offered to complete multiple pages of this task (each page containing 60 numbers that need to be screened) and were informed that we will plant one tree for each completed page (with a maximum potential of planting 8 trees; see Fig. 1E for the distribution).

#### Demographic predictors

##### Age

“How old are you?”. Single-line text box where the content type was restricted to numerical text (in years).

##### Gender

“What is your gender?”. Multiple choice (one answer): “Male”, “Female”, “Prefer not to say”, “Non-binary/third gender/other”. Due to the requirements of some internal review boards, this item may have been altered for the data acquisition of specific countries (https://osf.io/qbe84 for an overview).

##### Education

“How many years of formal education have you completed?” Multiple choice (one answer): “0-6 (up to grade school)”, “7-12 (up to high school)”, “13-16 (college/undergraduate degree/certificate training)”, “More than 17 years (doctorate degree, medical degree, etc.)”.

##### Political orientation

Participants were asked “What is your political orientation for…” (1) “social issues (e.g., health care, education, etc.)” and (2) “economic issues (e.g., taxes)”. Measured on a scale from 0 to 100 points from extremely liberal/left wing to extremely conservative/right wing. The two items were highly correlated and formed a single scale with higher scores indicating more political conservatism (α = 0.84).

##### Income

“What is your total yearly family/household income?”; income listed in 8 steps. I prefer not to say it was also listed as a possible response.

##### MacArthur’s subjective social status

“Please choose the rung where you think you stand at this time in your life relative to other people in your country”. Measured on a ladder with 10 steps (1-10).

For the distributions of all predictors, see SI Figure S1.

#### Psychological predictors

##### Trust in climate science

Two items; “On average, how competent are climate change research scientists?” and “On average, how much do you trust scientific research about climate change?” (α = 0.91). Slider from 0-100 from “Not at all” to “Very much so”. Participants were also allowed to respond with “No opinion”.

##### Trust in government

“On average, how much do you trust your government?”. Measured on a slider from 0 to 100, from “Not at all” to “Very much so”. Participants were also allowed to respond with “No opinion”.

##### Humanitarian identity

“To what degree do you see yourself as someone who cares about human welfare?”. Measured on a slider from 0 to 100 points from “Not at all” to “Very much so”. Participants were also allowed to respond with “No opinion”.

##### Global citizen identity

“To what degree do you think of yourself as a global citizen?”. Measured on a slider from 0 to 100 points, from “Not at all” to “Very much so”. Participants were also allowed to respond with “No opinion”.

##### Environmental identity

Four items adapted from prior work (α = 0.91)^57^, for example, “To what degree do you see yourself as someone who cares about the natural environment?” (α = 0.91). Measured with a slider from 0-100 from “Not at all” to “Very much so”.

##### Environmental motivation: internal

Adapted from prior work (α = 0.89)^58^. Five items, e.g., “I attempt to behave pro-environmentally because it is personally important to me.” (α = 0.89). Measured on a slider from 0 to 100 points from “Not at all” to “Very much so”.

##### Environmental motivation: external

Five items, e.g., “Because of today’s politically correct standards, I try to appear pro-environmental.” (α = 0.73). Adapted from prior work^58^.

Measured on a scale from 0 to 100 points from “Not at all” to “Very much so”.

##### Perceived climate change belief

“What percentage of people in your country do you think would agree with the statement *Climate change is a global emergency?*”. Measured on a slider from 0-100 with the label “% Who Agree”.

##### Perceived scientific consensus on climate change

“To the best of your knowledge, what percentage of climate scientists have concluded that human-caused climate change is happening?”. Slider scale from 0 to 100, measuring the estimate in percentage.

For the full set of items used for measuring these predictors and their distributions, see SI Figure S1.

#### Nation-level predictors

##### GINI coefficient

Income inequality index, which measures the extent to which the distribution of income or consumption among individuals or households deviates from a perfectly equal distribution. We sourced the most recent GINI coefficient for each country from https://data.worldbank.org/indicator/SI.POV.GINI.

##### HDI

Index combining life expectancy, education, and per capita income indicators, which is used to rank countries in their level of human development. We sourced the most recent HDI index for each country from https://ourworldindata.org/human-development-index.

##### Climate risk index

The Global Climate Risk Index quantifies the impacts of extreme weather events on nations worldwide. We sourced the data from The Global Climate Risk Index 2020 report, which analyzes fatalities and economic losses from climate-related disasters over a 20-year period (2000-2019). We retrieved this information from https://www.preventionweb.net/publication/global-climate-risk-index-2020#:~:text=The%20Global%20Climate%20Risk%20Index,2018%20%E2%80%94were%20taken%20into%20account. Important: Higher values on this index represents lower risk.

##### Carbon emissions per capita

The combination of emissions from fossil fuels and industry from each nation divided by total population. This measure is a ‘production-based’ measure of per capita CO_2_ emissions that does not account for imported fossil fuels. We sourced these data from https://ourworldindata.org/co2-emissions#per-capita-co2-emissions focusing on per capita CO2 emissions for the year 2020 measured in tons.

This is an already reduced set of nation-level predictors (see the preregistration for selection procedure - https://osf.io/6jc85). Before we ran the analysis, we also gathered data on GDP per capita, corruption perception index, and democracy index. However, correlations between GDP, corruption perception index, democracy index, and HDI were above 0.85. Thus, to avoid issues with the interpretability of such highly correlated predictors that potentially share predictive importance, we decided to keep only one of them - HDI - as it combines multiple indicators.

### Analysis

#### Exclusions

Data from eight countries from the published dataset were excluded due to systematic missing values (i.e., countries had above 80% missing values for some variables). Kenya, Tanzania, and Uganda were excluded as they did not collect data on political orientation due to restrictions from their ethics boards. Taiwan was excluded due to a lack of data for the climate risk index, HDI, and GINI. Singapore, Saudi Arabia, and New Zealand were excluded due to a lack of GINI data. We also removed Chile, as it had several variables with >80% missing data.

#### Machine learning approach

To analyze the data, we adopted an interpretable machine learning-based approach for multivariable statistical analysis, moving away from traditional methods. We opted for this approach as it can address several limitations associated with conventional statistical techniques^28^. Central to machine learning is its focus on predictive accuracy, achieved through out-of-sample inference. This process involves generalizing findings to new, unseen data using models that are flexible, complex, and high-dimensional but still remain robust and credible. This contrasts with the in-sample inference predominant in classical statistical methods^27–30,59,60^. Moreover, this machine learning-based analysis is not dependent on restrictive assumptions about variable interactions, variable scaling, or model oversimplification that often biases traditional statistical methods^27–29^. Therefore, machine learning offers a more robust and comprehensive approach to exploring complex datasets^27–30,59,60^. The multivariable statistical analysis we used is rooted in machine learning and draws inspiration from the methodology outlined by previous research^26^. It is conducted using Python v3.11.5 with the scikit-learn library v1.3.0^54^ and comprises three integral components: (1) the development of a prediction model, (2) assessment of its generalizability, and (3) model interpretation.

#### Prediction model development

For the prediction models, we selected Gradient Boosted Decision Tree (GBDT) models due to their computational efficiency and high accuracy^61,62^. GBDT models inherently capture non-linear associations and variable interactions^26^ and select the most informative predictor for splitting at each step. Importantly, they are robust against outliers and multicollinearity in the data. To prevent the dominance of one predictor over another in the case of highly correlated predictors, there are several built-in functionalities^62^. First, the sequential nature of tree building in GBDTs helps capture information left by correlated predictors not selected initially. Second, due to random predictor selection in each boosting round, some of the predictors are omitted regularly, and the correlated predictor is used. Third, L2 norm regularization in each leaf node helps to reduce the influence of one predictor and allows the use of the correlated predictor as well. We ran four separate models for each of the four climate-relevant outcome variables. The predictors for these models included the individual and nation-level variables (see Table 1), with each of the four outcomes serving as the prediction target.

#### Assessment of generalizability

To evaluate the models’ ability to generalize to unknown (out-of-sample) data, we implemented a nested cross-validation (CV) procedure, as recommended^31^. This procedure involves repeated splits of the data into training and testing sets. In the outer CV loop, a 10 times 5-fold split scheme grouped by participants was applied. Model complexity tuning, carried out in a nested (inner) CV procedure using training data only (5-folds repeated until a minimum of 1000 predictions), utilizes a random search scheme to identify optimal complexity parameters (column sample per tree 0.1 to 1; using extra trees True/False; path smoothing 1 to 1000 log-scale). The selected parameters are then employed in the main CV loop along with constant parameters (learning rate: 0.01; number of leaves: 100; number of boosting rounds: 1000; max bin: 100) for model training and testing. Regression performance is measured using the prediction coefficient of determination (prediction R²) and mean absolute error, while classification performance is evaluated using balanced classification accuracy.

#### Analysis of the variable’s predictive importance

Analysis of single predictors’ predictive importance was performed using SHAP (SHapely Additive explanations), a method from interpretable machine learning based on Shapley values from cooperative game theory^25^. SHAP measures the contributions of each predictor, considering interaction effects, for the prediction task, providing a comprehensive assessment of their importance. The SHAP value represents the difference between the model’s prediction with and without the predictor for a given instance. It quantifies how much the presence of a predictor shifts the model’s output compared to the average prediction. The importance of a predictor is defined as the mean absolute SHAP value across all instances. This reflects the total contribution of the predictor to the model’s predictions in terms of magnitude, regardless of the direction of influence (positive or negative). A higher mean absolute SHAP value indicates a greater impact on the model’s predictions. By aggregating SHAP values, one can rank predictors in terms of their relative importance, with higher SHAP values indicating predictors that have a more substantial influence on the model’s outcome.

#### Statistical significance

The assessment of statistical significance for differences between the means of prediction R² metric, prediction accuracy, and the importance of predictors was carried out using a modified t-test. It compares the observed values with their counterparts generated under a simulated null hypothesis, where data labels are subjected to shuffling. Notably, to address sample dependence introduced by cross-validation, the t-test is adapted, aligning with the recommendations^63^.

#### Deviations from the preregistration

We have not deviated from the preregistration in terms of the planned and conducted analysis (https://osf.io/6jc85). However, when presenting the main findings, we opted for grouping the predictors in such with consistent vs inconsistent effects across all outcome measures, which had not been specified in the preregistration. The intention of this way of grouping was to reduce the complexity of the large number of effects and help the reader interpret the findings more easily.

## Supplementary information

Supplementary Information

## Data Availability

The data used in this paper are publicly available on OSF: https://osf.io/m5uw7/files/osfstorage.

## Code availability

The code used for analyzing the data are publicly available on OSF: https://osf.io/m5uw7/files/osfstorage.
