# Supplementary material for: Machine learning identifies key individual and nation-level factors predicting climate-relevant beliefs and behaviors
Source: NPJ Clim Action. 2025 May 8;4(1):46. doi: 10.1038/s44168-025-00251-4 (PMC12061775; doi:10.1038/s44168-025-00251-4)
Supplement: Supplementary file 1 — Supplementary Information [file 44168_2025_251_MOESM1_ESM.pdf]

Supplemental information

Machine learning identifies key individual and nation-level factors predicting climate-relevant beliefs and behaviors

Boryana Todorova, David Steyrl, Matthew Hornsey, Samuel Pearson, Cameron Brick, Florian Lange, Jay Van Bavel, Madalina Vlasceanu, Claus Lamm, Kimberly Doell

Supplemental methods:

Questionnaire items for the outcome measures:

Climate change belief: Four items on a scale 0 = Not at all accurate to 100 = Extremely accurate (*M* = 77.36 , *SD* = 24.74, α = .93).

1. *Taking action to fight climate change is necessary to avoid a global catastrophe*
2. *Human activities are causing climate change*
3. *Climate change poses a serious threat to humanity*
4. *Climate change is a global emergency.*

Climate policy support: Nine items on a scale from 0 = Not at all to 100 = Very much so (M = 68.46 , SD = 19.79 , α = .88). There was also an not applicable option..

1. *I support raising carbon taxes on gas/fossil fuels/coal*
2. *I support significantly expanding infrastructure for public transportation*
3. *I support increasing the number of charging stations for electric vehicles*
4. *I support increasing the use of sustainable energy such as wind and solar energy*
5. *I support increasing taxes on airline companies to offset carbon emissions*
6. *I support protecting forested and land areas*
7. *I support investing more in green jobs and businesses*
8. *I support introducing laws to keep waterways and oceans clean*
9. *I support increasing taxes on carbon-intensive foods (for example, meat and dairy)*

Questionnaire items for the psychological predictors

Trust in climate science

Two items:

1. "*On average, how competent are climate change research scientists*?"
2. "*On average, how much do you trust scientific research about climate change?*"

Slider from 0-100 from "Not at all" to "Very much so". Participants were also allowed to respond with "No opinion". α = .91, *M* = 70.40, *SD* = 21.89

Trust in government

1. "*On average, how much do you trust your government?*".

Measured on a slider from 0 to 100, from "Not at all" to "Very much so". Participants were also allowed to respond with "No opinion". *M* = 44.28, *SD* = 28.64

Humanitarian identity

1. "*To what degree do you see yourself as someone who cares about human welfare?*".

Measured on a slider from 0 to 100 points from "Not at all" to "Very much so". *M* = 73.75.28, *SD*= 21.06

Global citizen identity

1. "*To what degree do you think of yourself as a global citizen?*"

Measured on a slider from 0 to 100 points, from "Not at all" to "Very much so". Participants were also allowed to respond with "No opinion". *M* = 67.80, *SD* = 25.18

Environmental identity

Four items adapted from prior work (α = .91)[1]: Measured with a slider from 0-100 from "Not at all" to "Very much so". *M* = 69.97, *SD* = 22.41

To what degree…

1. *do you see yourself as someone who cares about the natural environment*
2. *are you pleased to be someone who cares about the natural environment*
3. *do you feel strong ties with others who care about the natural environment*
4. *do you identify with others who care about the natural environment*

Environmental motivation: internal: 5 items, adapted from prior work (α = .89)[2]. Participants were asked to rate the degree to which they agree/disagree with the following statements about themselves:

1. *I attempt to behave pro-environmentally because it is personally important to me.*
2. *According to my personal values, acting non-environmental is OK. {reversed}*
3. *I am personally motivated by my beliefs to be pro-environmental.*
4. *Because of my personal values, I believe that acting anti-environmental is wrong.*
5. *Being pro-environmental is important to my self-concept.*

Measured on a slider from 0 to 100 points from "Not at all" to "Very much so". *M* = 69.97, *SD* = 20.52

Environmental motivation: external: 5 items, e.g. "Because of today's politically correct standards, I try to appear pro-environmental." (α = .73) Adapted from prior work[2].

1. *Because of today's politically correct standards, I try to appear pro-environmental.*
2. *I try to hide my negative thoughts about pro-environmental behavior in order to avoid negative reactions from others.*
3. *If I acted anti-environmental, I would be concerned that others would be angry with me.do you feel strong ties with others who care about the natural environment*
4. *I attempt to appear pro-environmental in order to avoid disapproval from others*
5. *I try to act pro-environmental because of pressure from others.*

Measured on a scale from 0 to 100 points from "Not at all" to "Very much so". *M* = 33.10, *SD* = 26.22

Perceived climate change belief:

1. "*What percentage of people in your country do you think would agree with the statement "Climate change is a global emergency?"*".

Measured on a slider from 0-100 with the label "% Who Agree". *M* = 60.03, *SD* = 21.90

Perceived scientific consensus on climate change:

1. *"To the best of your knowledge, what percentage of climate scientists have concluded that human-caused climate change is happening?"*

Slider scale from 0 to 100, measuring the estimate in percentage. *M* = 72.52, *SD* = 21.05

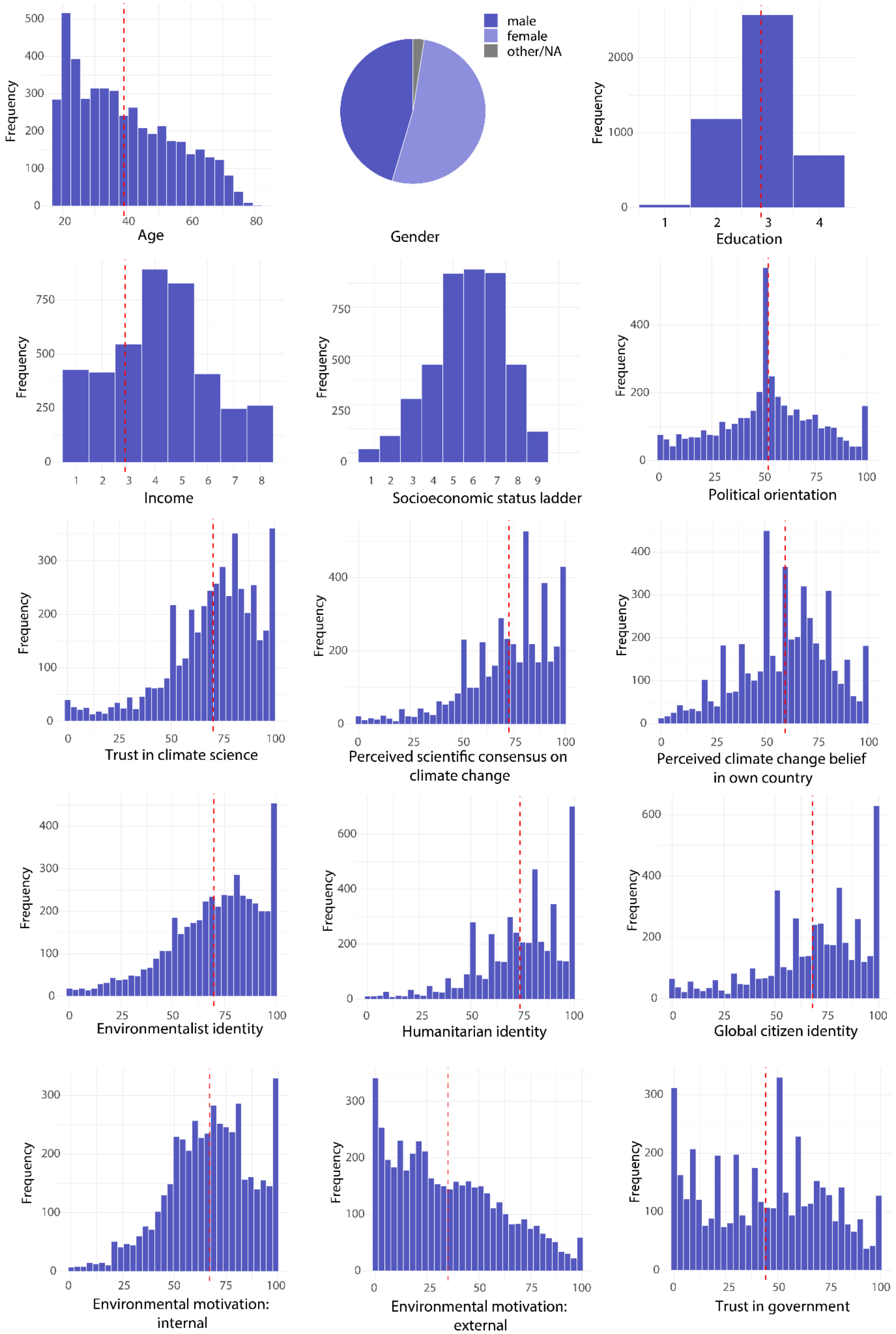

Figure S1. Distribution of the scores for the demographic and psychological predictors. Red dotted line indicates the mean

country	*n*	Mean age	SD age	Age lowest	Age highest	% male	% female
algeria	42	32.93	10.25	19	58	73.81	26.19
armenia	44	29.75	13.25	18	68	31.82	68.18
australia	81	46.26	16.50	18	73	47.50	52.50
austria	49	34.67	13.09	20	72	44.90	55.10
belgium	91	46.43	15.22	18	72	46.99	53.01
brazil	100	40.25	14.02	18	71	51.52	48.48
bulgaria	61	41.31	14.50	18	72	59.02	40.98
canada	103	28.31	16.25	18	74	28.00	72.00
china	77	27.82	6.77	19	57	32.47	67.53
czechia	52	27.81	13.54	18	69	30.77	69.23
denmark	78	46.17	17.48	19	79	48.98	51.02
ecuador	51	31.90	11.51	18	70	50.00	50.00
finland	129	38.81	14.41	19	73	34.38	65.63
france	116	49.77	15.39	18	74	51.30	48.70
gambia	43	25.60	7.20	18	50	43.90	56.10
germany	125	45.63	15.51	19	73	44.80	55.20
ghana	43	30.47	7.71	18	52	60.47	39.53
greece	63	40.34	11.83	18	75	53.23	46.77
india	60	27.83	9.97	18	63	63.79	36.21
ireland	65	28.15	10.49	18	58	51.56	48.44
israel	117	38.62	12.62	18	64	55.17	44.83
italy	132	36.20	14.41	18	71	52.03	47.97
japan	126	46.26	11.79	22	77	59.68	40.32
latvia	48	50.63	13.02	23	74	27.66	72.34
mexico	42	36.88	11.65	18	61	52.38	47.62
morocco	49	32.96	9.93	18	53	65.31	34.69
netherlands	152	38.80	18.67	18	79	36.24	63.76
nigeria	132	34.24	10.52	18	65	60.00	40.00
north macedonia	77	26.91	12.44	18	62	31.88	68.12
norway	84	52.07	14.19	21	78	54.88	45.12
peru	40	23.90	8.17	18	54	37.50	62.50
philippines	15	27.00	4.94	19	36	21.43	78.57
poland	199	41.37	16.79	18	82	46.70	53.30
portugal	42	26.45	8.66	19	51	47.62	52.38
romania	30	40.17	14.59	20	69	60.00	40.00
russia	116	29.79	11.84	18	65	38.05	61.95
serbia	33	42.63	15.51	18	65	29.03	70.97
slovakia	84	42.04	15.47	21	76	43.90	56.10
slovenia	38	34.57	10.67	18	63	51.35	48.65
south africa	39	36.51	12.76	18	64	58.97	41.03
south korea	54	44.11	13.40	18	70	50.00	50.00
spain	44	42.86	15.06	19	73	38.64	61.36
sri lanka	23	25.35	5.45	18	36	78.26	21.74
sudan	55	31.56	7.02	18	51	52.73	47.27
sweden	189	42.33	15.94	18	74	45.21	54.79
switzerland	95	42.73	14.82	18	77	43.16	56.84
thailand	49	37.84	10.18	19	66	59.18	40.82
turkey	62	32.84	12.75	18	66	28.33	71.67
uk	153	41.16	15.82	18	72	36.91	63.09
ukraine	46	33.73	13.07	18	60	32.56	67.44
united arab emirates	48	36.28	9.00	20	63	58.33	41.67
uruguay	39	37.03	12.15	19	59	25.64	74.36
usa	669	45.48	16.27	18	81	49.00	51.00
venezuela	7	53.29	10.03	35	68	57.14	42.86
vietnam	34	21.26	3.65	18	34	26.47	73.53

Table S1. Descriptives of each national sample

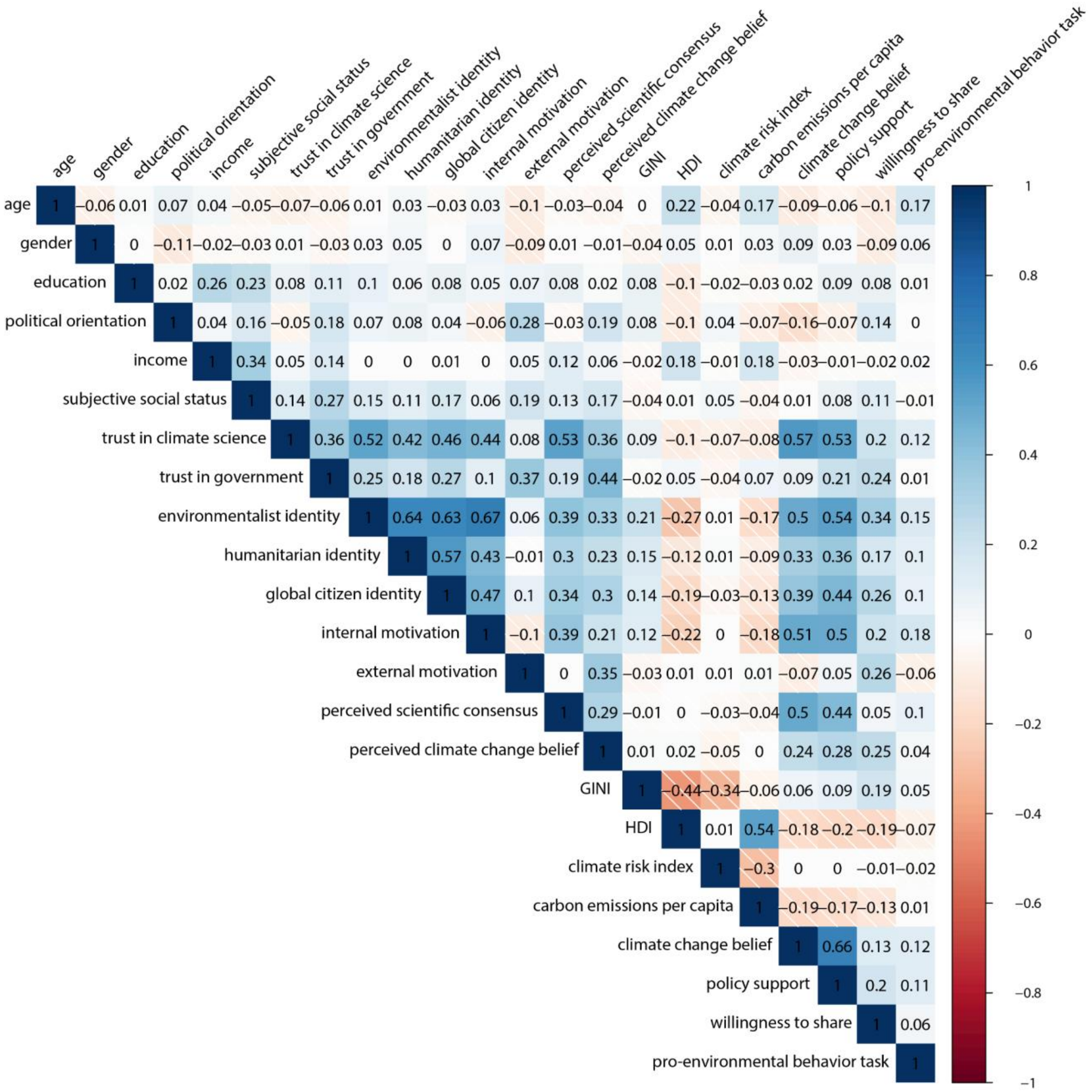

Figure S2. Correlation matrix (Pearson's correlation) of all predictors and outcomes included in the analysis.

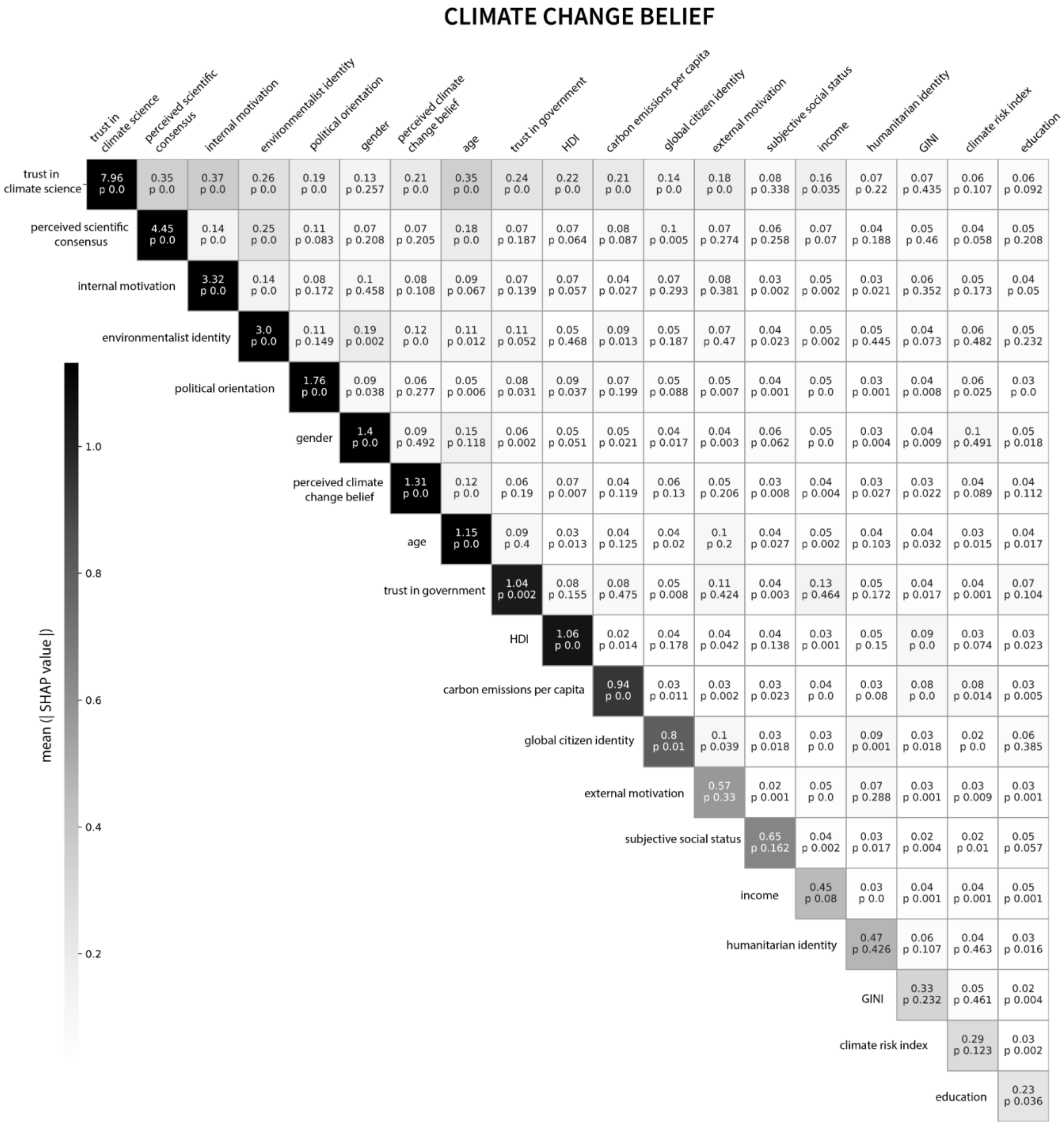

Figure S3. First level interactions for the model predicting climate change belief. The upper values in each box represent the mean absolute SHAP value (the higher it is, the bigger the effect of the respective interaction), while the lower value is the uncorrected P value (Pearson’s correlation). P values displayed as p 0.0 stand for *p* < 0.001. A plot of each individual interaction can be found on OSF in the first level interaction folder: https://osf.io/m5uw7/files/osfstorage#

POLICY SUPPORT

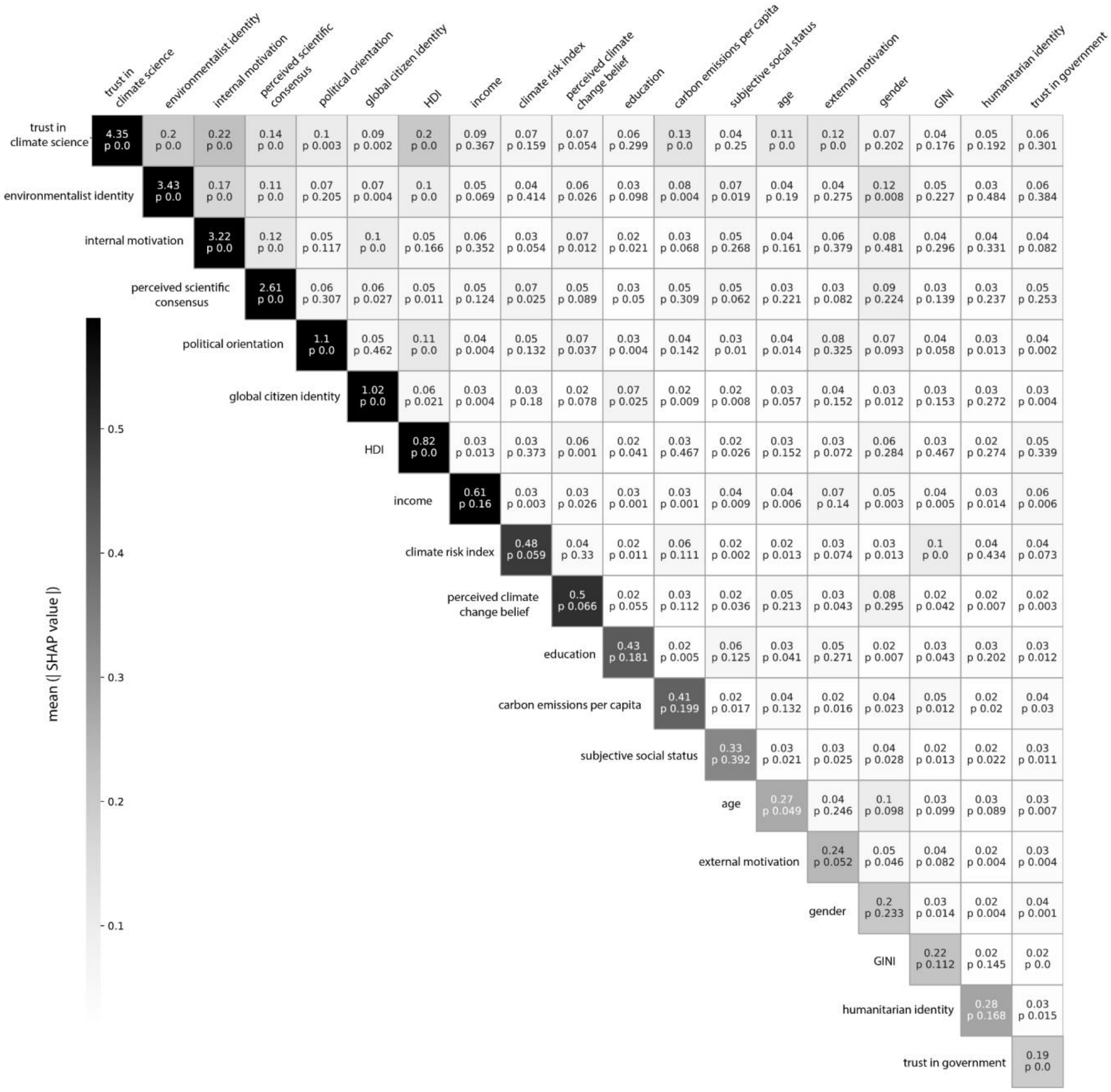

Figures S4. First level interactions for the model predicting climate policy support. The upper values in each box represent the mean absolute SHAP value (the higher it is, the bigger the effect of the respective interaction), while the lower value is the uncorrected P value (Pearson’s correlation). P values displayed as p 0.0 stand for $p < 0.001$. A plot of each individual interaction can be found on OSF in the first level interaction folder: https://osf.io/m5uw7/files/osfstorage#

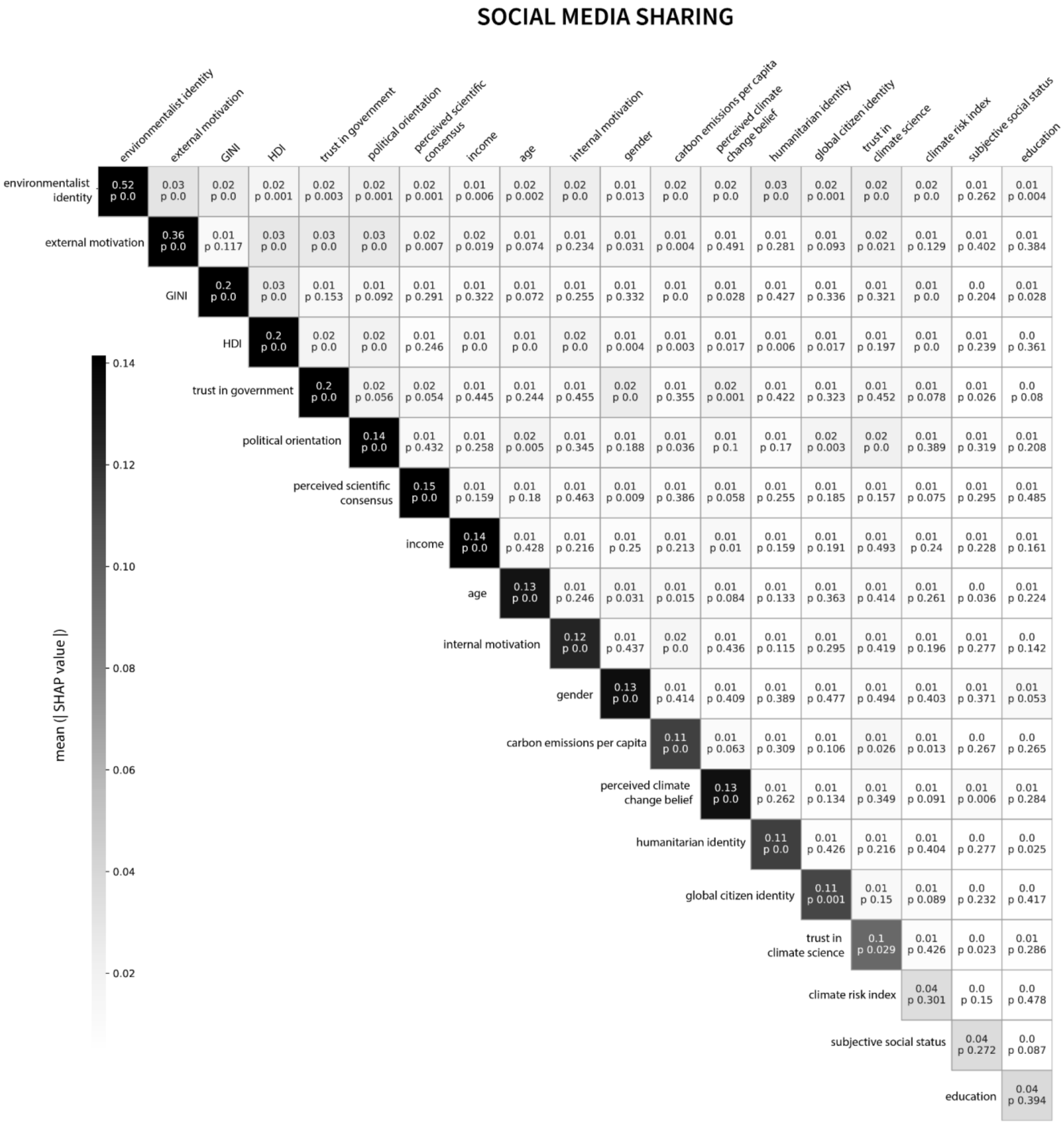

Figures S5. First level interactions for the model predicting willingness to share on social media. The upper values in each box represent the mean absolute SHAP value (the higher it is, the bigger the effect of the respective interaction), while the lower value is the uncorrected P value (Pearson's correlation). P values displayed as p 0.0 stand for *p* < 0.001. A plot of each individual interaction can be found on OSF in the first level interaction folder: https://osf.io/m5uw7/files/osfstorage#

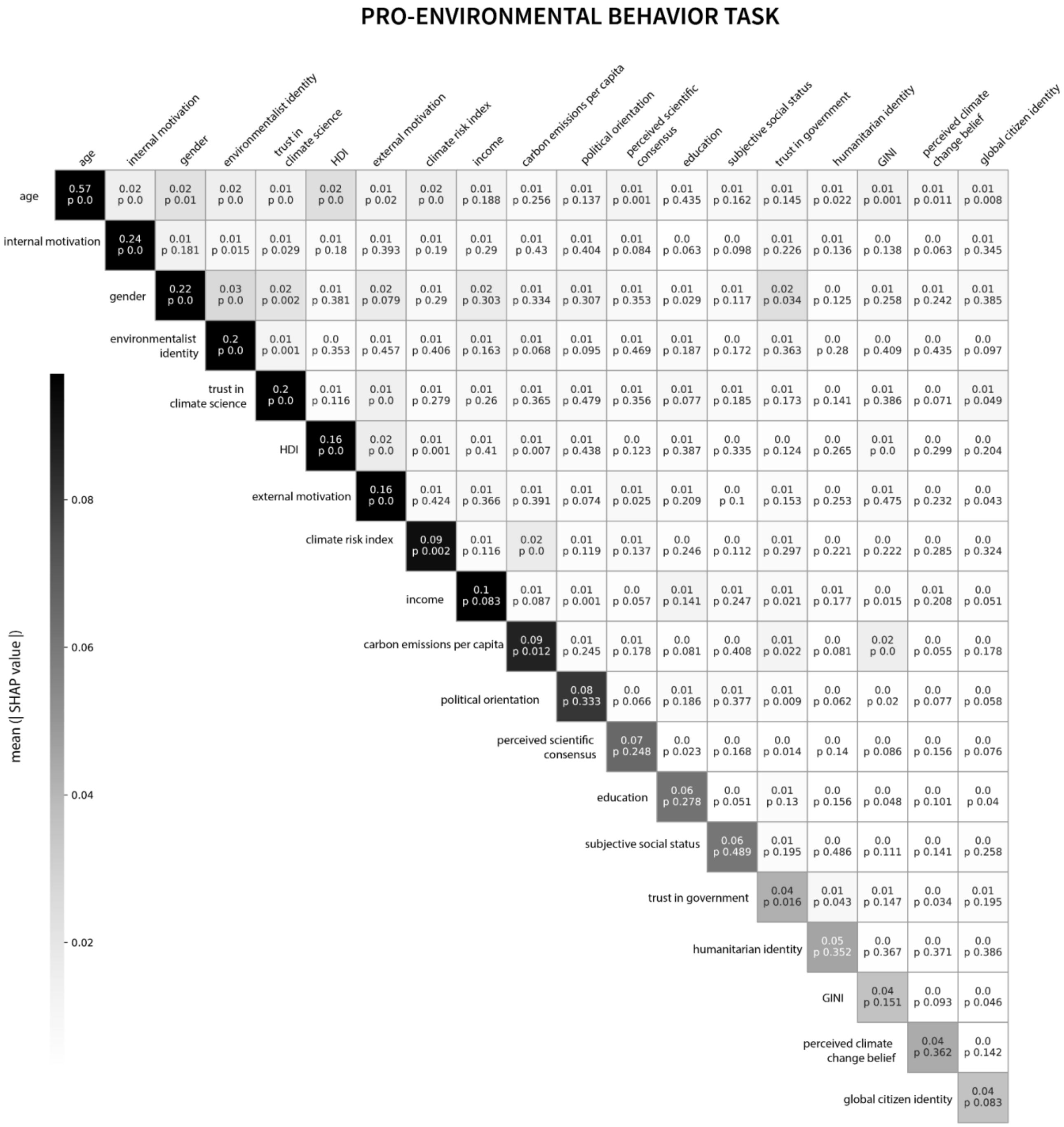

Figures S6. First level interactions for the model predicting the number of completed WEPT pages. The upper values in each box represent the mean absolute SHAP value (the higher it is, the bigger the effect of the respective interaction), while the lower value is the uncorrected P value (Pearson's correlation). P values displayed as p 0.0 stand for $p < 0.001$. A plot of each individual interaction can be found on OSF in the first level interaction folder: https://osf.io/m5uw7/files/osfstorage#

References

1. Brick, C., Sherman, D. K. & Kim, H. S. “Green to be seen” and “brown to keep down”: Visibility moderates the effect of identity on pro-environmental behavior. *Journal of Environmental Psychology* **51**, 226–238 (2017).
2. Brick, C. & Lai, C. K. Explicit (but not implicit) environmentalist identity predicts pro-environmental behavior and policy preferences. *Journal of Environmental Psychology* **58**, 8–17 (2018).
